# Cells´ Flow and Immune Cell Priming under alternating g-forces in Parabolic Flight

**DOI:** 10.1038/s41598-019-47655-x

**Published:** 2019-08-02

**Authors:** D. Moser, S. J. Sun, N. Li, K. Biere, M. Hoerl, S. Matzel, M. Feuerecker, J.-I. Buchheim, C. Strewe, C. S. Thiel, Y. X. Gao, C. Z. Wang, O. Ullrich, M. Long, A. Choukèr

**Affiliations:** 1 Laboratory of Translational Research “Stress and Immunity”, Department of Anaesthesiology, University Hospital, LMU Munich, Munich, Germany; 2 Key Laboratory of Microgravity (National Microgravity Laboratory), Center of Biomechanics and Bioengineering, and Beijing Key Laboratory of Engineered Construction and Mechanobiology, Institute of Mechanics, Chinese Academy of Sciences, Beijing, 100190 China; 3 School of Engineering Sciences, University of Chinese Academy of Sciences, Beijing, 100049 China; 4 Institute of Anatomy, Faculty of Medicine, University of Zurich, Zurich, Switzerland; 5 Department of Machine Design, Engineering Design and Product Development (IMK), Otto-von-Guericke-University Magdeburg, Magdeburg, Germany

**Keywords:** Inflammation, Innate immunity

## Abstract

Gravitational stress in general and microgravity (µg) in particular are regarded as major stress factors responsible for immune system dysfunction in space. To assess the effects of alternating µg and hypergravity (hyper-g) on immune cells, the attachment of peripheral blood mononuclear cells (PBMCs) to adhesion molecules under flow conditions and the antigen-induced immune activation in whole blood were investigated in parabolic flight (PF). In contrast to hyper-g (1.8 g) and control conditions (1 g), flow and rolling speed of PBMCs were moderately accelerated during µg-periods which were accompanied by a clear reduction in rolling rate. Whole blood analyses revealed a “primed” state of monocytes after PF with potentiated antigen-induced pro-inflammatory cytokine responses. At the same time, concentrations of anti-inflammatory cytokines were increased and monocytes displayed a surface molecule pattern that indicated immunosuppression. The results suggest an immunologic counterbalance to avoid disproportionate immune responses. Understanding the interrelation of immune system impairing and enhancing effects under different gravitational conditions may support the design of countermeasures to mitigate immune deficiencies in space.

## Introduction

It is almost six decades ago, that the first human was sent to space. Initially driven by political and technological prestige, curiosity and the desire of expanding knowledge about Earth and our solar system, human space flight nowadays maintains an important role in exploring the biology of life, the gravitational pull, and the lack thereof.

Another main task of space research is to assess the feasibility of long-term manned deep space missions, such as travelling to Mars. Besides technical aspects, physiological challenges for astronauts and the effects of adaptation to it remain a huge hurdle. During space missions, microgravity (µg) is considered to be one of the major stress factors responsible for dysfunctions of the innate and adaptive immunity^1^. The kind of immunological dysfunction varies strongly among different individuals and may lead either to hypo-reactivity, resulting in increased viral (re-) activation and susceptibility to infection^2,3^ or hyper-reactivity leading to hypersensitivity reactions like allergies or autoimmunities^3–5^. In µg, leukocytes display disturbed functions^6^ such as upregulated production of nitric oxides and of pro-inflammatory interleukins (IL) 6 and 8^7^. The ability of monocytes and macrophages to produce *reactive oxygen species* (ROS)^8,9^ is impaired in µg which is attributed to diminished *spleen tyrosine kinase* (Syk)-signalling^9^. Moreover cytokine secretion (IL-1) and recognition (IL-2 receptor) were reported to be also affected^10^. In T-cells, µg causes an impairment of activating signalling pathways including *protein kinase C* (PKC), *nuclear factor ‘kappa-light-chain-enhancer’ of activated B-cells* (NF-κB) and *mitogen-activated protein kinase* (MAPK)^10,11^. Together with a reduction of IL-2 receptor and *cluster of differentiation* (CD)3^12^ expression as well as cell cycle progression^13^, µg leads to a diminished activation of T cells.

Furthermore, a reduced expression of surface-bound molecules like *intercellular adhesion molecule 1* (ICAM1) is thought to contribute to impaired cell migration and activation of cells from both the innate and adaptive immune system^14^.

Hypergravity (hyper-g) on the other hand, which occurs during launch of space vehicles and landing back on Earth, stimulates immune responses. Macrophages produce higher amounts of ROS after stimulation^8^ and lymphocytes display higher activation levels in response to mitogens^15^.

Aside from this, changes in gravity affect the organization of cytoskeletal proteins which orchestrate adaptation processes to mechanical stress^16–18^. Thus, permanent remodelling of the cytoskeleton by alternating µg and hyper-g conditions has also an effect on cell mechanical stability^19,20^, motility and adhesion^21^ and cell signalling^11^.

We aimed to assess the impact of alternating g-forces on the adhesion properties of immune cells and their functional capacity under immune-stimulation. For this we performed *in vitro* experiments in parabolic flight (PF).

## Results

### Set-up A: Flow-chamber experiments

**Figure 1:**
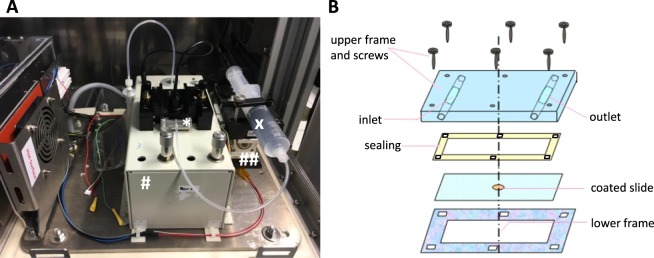
Experimental hardware and flow-chamber assembly. (**A**) Main experimental hardware in each incubator box was a syringe pump (##) in which a syringe prefilled with cell suspension (X) was installed. Cell suspension was pumped into a flow-chamber (*) and cells were then detected by a 40× objective and recorded by camera (#). (**B**) Flow-chamber assembly.

*Attaching cells and their rolling speed in different gravitational states* (*for set-up see* Fig. 1; *for representative* Videos 1 and 2, *see Supplementary Information*).

**Figure 2:**
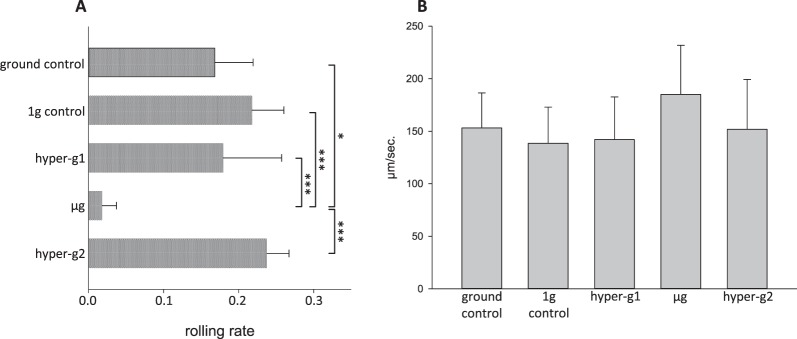
Rolling rate and rolling speed on adhesion molecule substrate. PBMCs were injected in a flow-chamber coated with ICAM1/PSGL-1 and floating/rolling behaviour was recorded. Analysis included video sequences from multiple parabolas. Time periods of videos and counted cell number were in a comparable range. hyper-g1: *pull-up*; hyper-g2: *pull-out* **(A)** Rolling rate of PBMCs at different gravitational conditions. **(B)** Rolling speed [µm/sec.] of rolling PBMCs at different gravitational conditions. Bar charts represent mean values of 10–20 parabolas or 273–752 (for **A**) or 6–119 (for **B**) cells with ± SD (*p < 0.05, ***p < 0.001).

In µg-periods, rolling rates of peripheral blood mononuclear cells (PBMCs) on ICAM1/*P-selectin glycoprotein ligand-1* (PSGL-1) substrates decreased significantly in comparison to ground control or 1 g control, respectively. In hyper-g (1.8 g), rolling rate was significantly higher than in µg with comparable values to controls (Fig. 2A). Cell number counts for all gravity conditions showed comparable levels (µg: 285, hyper-g1: 273, hyper-g2:316, 1 g: 326, ground control: 752).

Decreased rolling rate in µg was accompanied by a mild increase in average cell rolling speed (185 ± 47 µm/sec.) compared to control and hyper-g, which all showed similar velocities (1 g: 139 ± 34 µm/sec., ground control: 153 ± 33 µm/sec., hyper-g: 142 ± 41 and 152 ± 47 µm/sec). However, no statistically significant difference was reached between the velocities (rolling speed) under the different conditions (Fig. 2B).

### Set-up B: whole blood assays

*Activation patterns of monocytes and lymphocytes after 6 hours antigen-incubation.*

### Protocol “antigens during PF”

**Figure 3:**
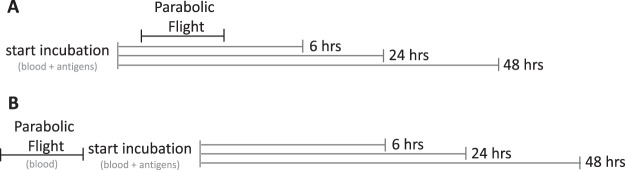
Schemes of different treatment protocols of PF and DTH assay. (**A)** Whole blood was mixed with antigens and exposed to a simultaneous PF in the early phase of antigen incubation: “antigens during PF”. **(B)** Whole blood was exposed to PF and subsequently incubated with antigens: “antigens after PF”. Total incubation time for DTH assays from both protocols was 6, 24 and 48 hours.

**Figure 4:**
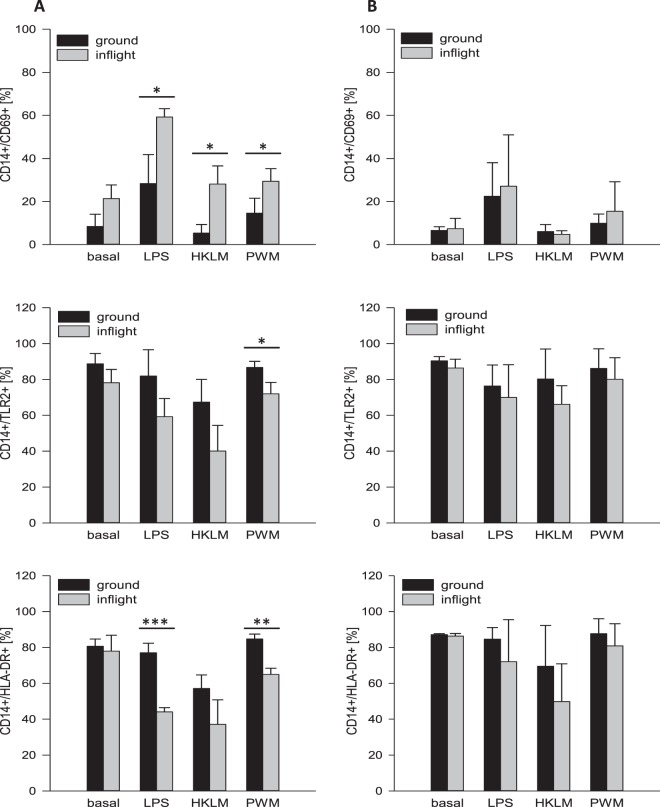
Expression of activation markers CD69, TLR2 and HLA-DR on monocytes after 6 hours of antigen incubation (**A)** protocol “antigens during PF” **(B)** protocol “antigens after PF”. After a total incubation time of 6 hours, samples were fixated for flow cytometric analysis of surface molecule expression. upper row: Double-staining of CD14 and CD69, middle row: Double-staining of CD14 and TLR2, bottom row: Double-staining of CD14 and HLA-DR. Bar charts represent mean values of double-positive cells as a percentage in samples from three different blood donors with ± SD (*p < 0.05, **p < 0.01, ***p < 0.001). Flow cytometry comprised of 10,000 events per sample.

Six hours of incubation with Lipopolysaccharide (LPS) resulted in a distinctly elevated expression of the activation marker CD69 on the cell surface of monocytes in comparison to basal control. CD69 expression did not or only moderately increase after single incubation with heat-killed *Listeria monocytogenes* (HKLM) or Pokeweed mitogen (PWM). Single exposure to PF led to increased CD69-levels in comparison to ground control, however, without reaching statistical significance. Antigen incubation during PF resulted in a significantly increased expression of CD69 in comparison to antigen incubation on ground, with the strongest increase in LPS-incubated samples (Fig. 4A, upper bar chart, for treatment protocol see Fig. 3A).

An opposite effect was observed for the expression of *Toll-like receptor* (TLR) 2 and *human leukocyte antigen* (HLA)-DR on monocytes. For TLR2, PF and single incubation with HKLM resulted in a mild decrease of surface expression. Combination of PF and antigens caused a moderate decrease of TLR2 in comparison to antigen treatment on ground, with PWM reaching statistical significance (Fig. 4A, middle bar chart). HLA-DR expression was reduced after single HKLM-incubation in comparison to basal control. Combined exposure to PF and LPS or PWM resulted in a highly significant decrease of HLA-DR on monocyte surface (Fig. 4A, bottom bar chart).

No differences were detected for CD40 and CD80 expression on monocytes (data not shown). Sample analysis after 24 hours incubation revealed a gradual reduction of the combination effect and an alignment of ground and inflight values (Table 1 and Figs. S1 and S2).

**Table 1:** Expression of activation markers CD69, TLR2 and HLA-DR on monocytes 24 hours after protocol “antigens during PF”.

antigens during PF		Basal		LPS		HKLM		PWM	
CD14^+^		mean	SD	mean	SD	mean	SD	mean	SD
CD69^+^	**ground**	21,23	±17,52	29,50	±5,32	16,09	±8,58	33,41	±11,34
	**inflight**	27,61	±8,98	47,7*	±5,78	23,17	±6,63	44,29	±9,45
TLR2^+^	**ground**	60,28	±14,74	51,47	±7,98	35,44	±6,96	55,34	±10,53
	**inflight**	52,85	±19,83	38,43	±5,07	25,69	±13,06	48,2	±8,46
HLA-DR^+^	**ground**	48,52	±9,30	49,47	±12,70	25,39	±5,56	52,75	±8,78
	**inflight**	45,47	±12,70	37,54	±2,03	23,31	±11,85	47,74	±9,56

After a total incubation time of 24 hours, samples were fixated for flow cytometric analysis of surface marker expression. Displayed are mean values of double-positive staining as a percentage (CD14/CD69, CD14/TLR2, CD14/HLA-DR) in samples from three different blood donors with ± SD (*p < 0.05).

Expression of CD28 and CD152 on CD4^+^ and CD8^+^ lymphocytes showed no changes under any of the conditions (data not shown).

### Protocol “antigens after PF”

Monocyte surface expression of CD69 and TLR2 from inflight samples showed similar expression levels to samples incubated on ground. Only HLA-DR expression was slightly affected by a combined exposure to PF and LPS or HKLM, respectively. However, effects were very low and did not reach statistical significance (Fig. 4B, data for incubation time 24 hours in Table 2 and Figs S3 and S4, for treatment protocol see Fig. 3B). Here again, expression of CD40 and CD80 on monocytes as well as CD28 and CD152 on CD4^+^ and CD8^+^ lymphocytes was unchanged (data not shown).

**Table 2:** Expression of activation markers CD69, TLR2 and HLA-DR on monocytes 24 hours after protocol “antigens after PF”. After a total incubation time of 24 hours, samples were fixated for flow cytometric analysis of surface marker expression. Displayed are mean values of double-positive staining as a percentage (CD14/CD69, CD14/TLR2, CD14/HLA-DR) in samples from three differe nt blood donors with ± SD.

antigens after PF		Basal		LPS		HKLM		PWM	
CD14^+^		mean	SD	mean	SD	mean	SD	mean	SD
CD69^+^	**ground**	18,56	±9,91	30,91	±17,64	13,28	±7,47	27,20	±18,09
	**inflight**	19,49	±8,10	31,05	±6,82	12,01	±6,79	28,88	±13,56
TLR2^+^	**ground**	62,93	±12,76	50,29	±12,47	35,68	±10,66	60,73	±13,18
	**inflight**	56,65	±18,12	48,09	±14,82	38,56	±8,91	62,72	±7,53
HLA-DR^+^	**ground**	50,73	±15,77	51,12	±13,60	28,06	±1,63	53,99	±7,49
	**inflight**	43,30	±22,95	45,63	±19,16	28,88	±10,18	50,39	±11,50

Between donors, percentages of lymphocyte and monocyte populations were within the normal range and did not vary significantly (lymphocytes 30.16% ± 5.32, monocytes 3.01% ± 0.92).

*Cytokine pattern after 6 hours antigen-incubation.*

### Protocol “antigens during PF”

**Figure 5:**
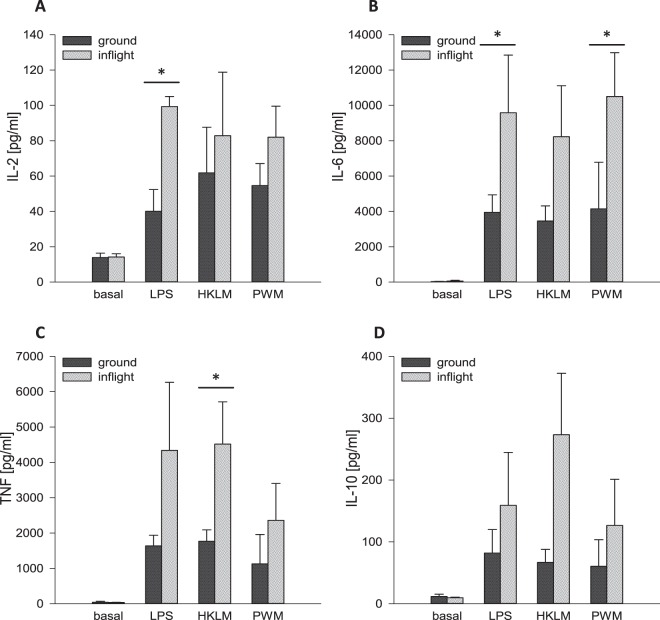
Cytokine expression after 6 hours of incubation at “antigens during PF”. After a total incubation time of 6 hours, plasma supernatants were collected for cytokine analysis of **(A)** IL-2 **(B)** IL-6 **(C)** TNF and **(D)** IL-10. Bar charts represent mean values of cytokine concentration [pg/ml] calculated from the flow cytometrically determined MFI of samples from three different blood donors with ± SD (*p < 0.05).

On ground, single incubation with all three antigens led to comparably elevated concentrations of the pro-inflammatory cytokines IL-2, IL-6 and *tumor necrosis factor* (TNF). PF alone did not alter cytokine concentration. Antigen incubation during PF resulted in a clear augmentation of these cytokines with a significant increase of IL-2 after LPS incubation, IL-6 after LPS and PWM incubation and TNF after HKLM incubation, respectively (Fig. 5A–C).

Concentration of the immune-suppressive cytokine IL-10 increased likewise in response to single incubation with all three antigens. Simultaneous PF induced an additional, but insignificant potentiation. PF alone had no effect on IL-10 levels (Fig. 5D).

Similar to the expression of activation markers on monocytes, elevation of cytokine levels displayed a limitation to early incubation time points. After 24 and 48 hours of incubation, values from ground and inflight samples gradually aligned with each other (data not shown).

### Protocol “antigens after PF”

**Figure 6:**
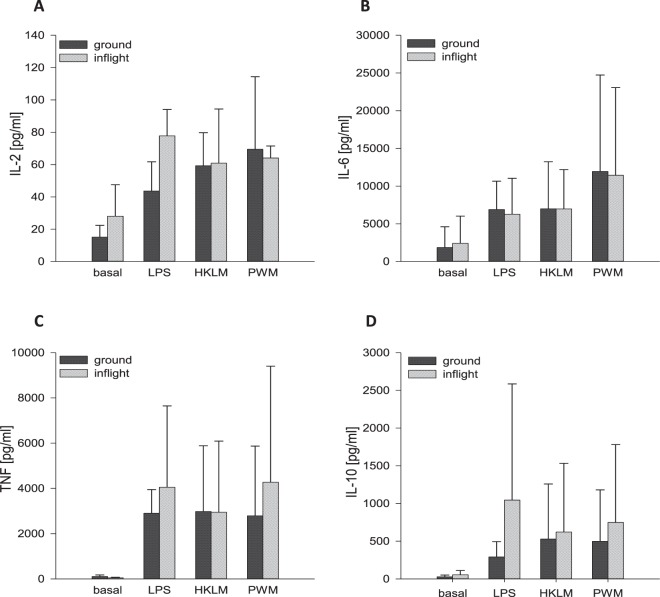
Cytokine expression after 6 hours of incubation at “antigens after PF”. After a total incubation time of 6 hours, plasma supernatants were collected for cytokine analysis of **(A)** IL-2 **(B)** IL-6 **(C)** TNF and **(D)** IL-10. Bar charts represent mean values of cytokine concentration [pg/ml] calculated from the flow cytometrically determined MFI of samples from three different blood donors with ± SD.

Antigen stimulation on ground induced an increase in cytokine concentration. However, PF prior to antigen incubation did not additionally affect cytokine levels (Fig. 6, 6 hours, data for 24 hours and 48 hours not shown).

## Discussion

The condition of µg is considered to be one of the major stress factors responsible for immune cell impairment during space flight^1,22–25^. In contrast, hyper-g, which occurs during starting and landing of space crafts, has a stimulatory effect on immune cell functions^8,15^.

In this study, we investigated *in vitro* the effects of acute gravitational stress in PF on primary immune cells.

During a µg-period, PBMCs display a reduced ability to adhere to adhesion molecules. Moreover, we demonstrated that alternating µg and hyper-g (1.8 g) conditions potentiate antigen-induced immune cell activation and cytokine secretion.

### Cell flow under g-forces

To our knowledge this is the first study that investigated the cell flow and adhesion of primary immune cells in a flow-chamber system during different gravitational conditions in PF. For coating of flow-chambers, we used the adhesion molecule ICAM1, which interacts with β2 integrin *lymphocyte function-associated antigen 1* (LFA-1) and *macrophage-1 antigen* (Mac-1) and PSGL-1, the major ligand of L-Selectin on endothelial cells and leukocytes^26,27^. Under physiological shear flow conditions, binding and subsequent rolling of activated leukocytes on these two receptors on the vascular endothelium are the first and indispensable steps for transendothelial migration from small blood vessels to inflammation sites^27–29^. We observed a moderate, however not significant acceleration of PBMCs´ flow and rolling speed in µg compared to hyper-g and 1 g phases. Binding to the adhesion molecule substrate was strongly affected in µg, which was mirrored by a significantly reduced rolling rate. In µg, prevailing lift forces mediate a centralization of flow and thus shifting of cells from the chamber walls to the middle of the flow-chamber^30^. Consequently, cells in close proximity to the adhesion molecule substrate are moved to the centreline flow, thereby making binding impossible and this presumably prevents proper activation^11^. At 1 g and especially at the transition to hyper-g, a rapid sedimentation of the cells occurs which in turn facilitates binding to the adhesion molecule substrate. To further analyse the impact of alternating gravitational conditions, remodelling of the cytoskeleton and changes at the transcriptional level should constitute a focus of future studies.

### Immune performance under g-forces

In the second part of the study, we investigated antigen-induced immune cell activation after acute gravitational stress by PF. For this, we performed whole blood (*delayed type hypersensitivity* (DTH)-) assays employing the bacterial antigens LPS and HKLM, which are ligands for TLR4 and 2, and PWM, which induces activation and mitosis in lymphocytes^31^.

For the *“antigens during PF”* protocol, single incubation with LPS resulted in a distinctly elevated expression of CD69 on monocytes, whereas incubation with HKLM or PWM had no or only minimal effects. Under physiological conditions, CD69 is expressed only by a small percentage of monocytes (approx. 10%). Stimulation with leptin, LPS or phorbol 12-myristate 13-acetate (PMA), however, has been shown to enhance CD69 levels^32^. Crosslinking of CD69 potently activates monocytes, resulting in the production of inflammation mediators and cytotoxicity^33^. Furthermore, CD69 on monocytes is functionally associated with 5-lipoxygenase which is crucial for conversion of arachidonic acid to leukotrienes, factors that are attributed to hypersensitivities and allergies^32,33^. PF alone likewise resulted in enhanced CD69 expression, which indicates a primed state of monocytes, leading to an increased sensitivity towards further immune challenging. Consistent with this, combined exposure to PF and LPS strongly potentiated CD69 expression after 6 hours incubation. A comparable “priming” of polymorphonuclear leukocytes was demonstrated by Kaufmann and colleagues^34^. They observed in *ex vivo* analyses, that leukocytes display enhanced cytotoxic capabilities after PF which they attributed to immune cell “priming”, leading to an increased susceptibility of immune cells to stimuli^34,35^. The cytokine expression patterns we observed in our study, further support the hypothesis of immune cell “priming” by PF and an associated reinforced immune response. Concentrations of the pro-inflammatory cytokines IL-2, IL-6 and TNF were clearly elevated after single antigen incubation and a simultaneous exposure to PF resulted in further increase, indicating an enhancement of cell-mediated immunity. However, data reached only occasionally statistical significance (see Fig. 5), although increase of cytokine concentration showed a comparable course in all blood donors (data not shown). This lack of significance is attributed to high inter-individual variabilities in cytokine levels, which allowed only few data sets to reach statistical significance. Cytokine levels were unaffected by PF alone, suggesting that the “priming” effect in this set-up is mainly restricted to the activation state of monocytes. This is further supported by the constant expression levels of the activation and exhaustion markers CD69, CD152 and CD28 on CD4^+^ and CD8^+^ lymphocytes, respectively.

Interestingly, immune enhancing effects were accompanied by cell surface molecule patterns indicating immunosuppression. In contrast to single antigen incubation, a simultaneous exposure to PF resulted in a reduced expression of TLR2, the major pattern recognition receptor on monocytes to recognize pathogens and to initiate their clearance, mainly by phagocytosis^36^. However, only a combination of PF and PWM induced statistically significant reduction. Incubation with LPS and PWM during PF resulted in a significantly decreased HLA-DR surface expression. HLA-DR is a component of the major histocompatibility complex class II and crucial for processing and presenting antigens from exogenous proteins to CD4^+^ lymphocytes^37–39^. Thus, surface expression level of HLA-DR represents an excellent marker for monocyte functionality and the cells´ anergy and a reduced expression correlates with decreased immune-responsiveness^39,40^. Physiological trauma was shown to be associated with a decreased expression of TLR2 and HLA-DR on monocytes in the first 5.5 hours after induction in a post-traumatic porcine model. The reduced expression of both surface molecules was accompanied by immune dysregulation, which was demonstrated by reduced phagocytic activity in this model^41^.

Our observation of a concomitant immune-suppressed state was further confirmed by elevated IL-10 levels. This anti-inflammatory cytokine is expressed during inflammation in parallel to pro-inflammatory cytokines and plays a decisive role in limiting host immune response to pathogens, thereby preventing damage to the host and maintaining normal tissue homeostasis^42,43^. Interestingly, IL-10 is capable of suppressing the expression of HLA-DR^38^ which may be another reason for the observed downregulation of this receptor on monocyte surface.

Taken together, PF induces a pronounced but only temporary “primed” state of monocytes, which increases the sensitivity towards a concomitant incubation with antigens and leads to a strong cell-mediated immune response. At the same time, anti-inflammatory factors are released to compensate for the strong pro-inflammatory state and to prevent inappropriate immune responses. Whether reduced expression levels of TLR2 and HLA-DR after combined treatment are a mechanism to induce compensatory immune-suppression or a symptom of it has yet to be elucidated in future studies.

To exclude a shift of monocyte subsets rather than monocyte “priming”, we analysed the proportion of CD14 and FCγIII receptor CD16^44^ on these cells. Percentage distribution of the classical monocyte subset (CD14^++^CD16^−^) with mainly phagocytic and antigen-presenting functions and the intermediate (CD14^++^CD16^+^) and non-classical (CD14^+^CD16^++^) phenotype with predominantly inflammatory functions^38,44^ was unaffected by PF (Fig. S5). This further corroborates our statement of monocyte “priming” by PF.

For the *“antigens after PF”* protocol, comprising PF exposure prior to antigen incubation, we observed no significant differences in CD69, TLR2 and HLA-DR expressions between ground and inflight samples. Cytokine concentrations were elevated after antigen incubation, but also here, an additional increase by PF was missing.

Therefore, we suggest that reinforced antigen-induced immune responses due to “priming” require an immediate additional stimulus. For the “antigens after PF” protocol, there was a time window of approximately one hour between PF and start of antigen-incubation (see Materials and Methods section) in which cells obviously recovered from gravitational stress leading to a lack of immune enhancement.

It has to be pointed out, that due to overall limitations of the entire set-up and low subject number, the conclusions drawn from the experiments have to be further validated in future studies. Single subject analysis revealed, that expression of cell surface markers (CD14, CD69, TLR2, HLA-DR) and cytokine secretion show comparable courses for all three subjects (data not shown), which makes these observations very much worthy of further follow-up.

## Conclusion

In µg, interactions between immune cells and adhesion molecules are impaired because of a centralization of flow, which may represent a contributing factor for immune system dysfunction under this condition. However, gravitational stress by PF modulates the activation pattern of immune cells *in vitro*, resulting in “priming” towards simultaneous immune challenging. These opposite observations indicate that potentiated immune responses after PF and abrogation of µg-mediated immune impairment are induced by hyper-g, which occurs at the *pull-up* (hyper-g1) and *pull-out* (hyper-g2) phase of each parabola. Further investigation of these interrelations may support the development of gravitational countermeasure options to mitigate such immune deficiencies.

## Materials and Methods

### Blood donors and blood storage

Blood was obtained from the local blood bank “*l’Etablissement français du sang*” (EFS) in Bordeaux, France from three different donors (two males, one female, median age: 51 years) through venous puncture of the forearm.

PBMCs for flow-chamber experiments (Set-up A) were isolated from *Citrate Phosphate Dextrose Adenine* (CPDA)-blood bags (400–450 ml), which were stored after blood draw at room temperature until PBMC-isolation on the same day. For whole blood assays (Set-up B), blood was stored at room temperature in lithium heparin tubes until start of incubation experiments on the following day.

### Induction of acute gravitational stress by parabolic flight

Experiments were performed during the 30^th^ DLR (*Deutsches Zentrum für Luft- und Raumfahrt*, German Aerospace Center) PF campaign (4–16 September 2017) at Novespace (Bordeaux, France). One parabola consists of a hyper-g-phase (1.8 g, *pull-up*, 22 s), which is followed by a short-term µg-period (22 s) and completed by an additional hyper-g-phase (1.8 g, *pull-out*, 22 s), resulting in acute gravitational stress^34^. One flight day consists of 31 parabolas.

### Set-up A: Flow-chamber experiments

The ability of PBMCs to attach to an adhesion molecule substrate under different gravitational conditions was investigated in a self-developed flow-chamber system at a visual level (Fig. 1).

#### Isolation of peripheral blood mononuclear cells and cultivation

PBMCs were isolated by Histopaque (Histopaque 1077, Sigma Aldrich, Steinheim, Germany) density gradient centrifugation using Leucosep tubes (Greiner, Kremsmuenster, Austria) and were subsequently seeded in Roswell Park Memorial Institute 1640 cell culture medium (RPMI, Sigma-Aldrich, Steinheim, Germany), supplemented with 10% fetal calf serum, 1% penicillin/streptomycin and incubated overnight at 37 °C with 5% CO_2_. On the experiment day, cell number and viability were determined (TC20 cell counter, BioRad, Hercules, CA, USA) and cells were transferred to 1% bovine serum albumin (BSA)/PBS into a 50 ml syringe and kept at 37 °C until installation into the experimental hardware.

#### Coating of slides, assembly of flow-chamber and experimental hardware

For coating, the surface of Nunc 160005 Permanox™ Microscope slides (Nunc, Thermo Scientific, Waltham, MA, USA) was pre-incubated (2 hours, 37 °C) with anti-human IgG secondary antibodies (200 µg/ml; Sigma-Aldrich, Steinheim, Germany) and subsequently blocked with 1% BSA/PBS (4 °C) overnight. Blocked slides were double-coated with ICAM1- and PSGL-1-IgG Fc chimeras (1 hour, 37 °C, both 5 µg/ml, from R&D Systems, Minneapolis, MN, USA). Coated slides were embedded into the flow-chambers and installed in the experimental hardware, which was assembled into incubator boxes in experimental racks (Fig. 1).

#### Experimental run and data recording

Immediately before take-off, syringes with cell suspension were installed in the automatic syringe pump device. Having reached the cruising altitude and approximately 10 minutes before start of PF, pumps were started and cell suspension was pumped into the flow-chamber. The flow rate was set at 0.4 ml/min, thus the calculated shear stress near the slide surface was 0.05 Pa, which lies in the physiological range of blood flow-induced shear stress in postcapillary venules^45^. A 40 × objective subassembly with a CCD camera (Wat-660D, Watec CO., LTD., Yamagata-Ken, Japan) was located near the inlet, which recorded passing cells. For ground controls, experiments were performed in the same setting under laboratory conditions.

#### Determination of cells rolling on adhesion molecule substrate

All cells that were fully visible in the video-sequences within a defined time period were counted. The rolling cells were determined from the video by their clearly slow moving speed (Supplementary Video 1). The rolling rate is calculated by the number of rolling cells divided by the total cell count. By this, proportion of rolling cells was derived. This procedure was implemented for all gravitational conditions.

#### Determination of rolling speed

Speed of rolling cells was analysed from the video imaging using the tracking module of NIS-ELEMENT software (Nikon, Tokyo, Japan). The rolling speed is calculated by the moving distance per time frame and then averaged over the time window passing the frames of vision.

### Set-up B: Whole blood assays

Immune cell activation and cytokine response was analysed after exposure of whole blood to PF and immune challenging by recall antigens and a mitogen.

Whole blood was subjected to a cytokine release assay mimicking elements of a DTH reaction as described before^46,47^ and is further mentioned as “DTH assay” in this paper.

For exposure to gravitational stress by PF, two different treatment protocols were designed. In the first one, DTH assay was set up on ground in the morning of the flight day and subsequently exposed to PF. After PF, the remaining incubation time until completion was carried out in incubators on ground (“antigens during PF”, Fig. 3A). The second protocol comprised of a single exposure of the whole blood sample-vials to PF and DTH assay was started afterwards (“antigens after PF”, Fig. 3B). For this treatment protocol, there was a time window of approximately one hour between the last flown parabola and start of antigen-incubation. Reasons for this were i) the time needed for return from the PF manoeuvre site back to the airport (30 minutes), ii) disassembly of the incubation chambers in the plane and transport of the flown blood to the laboratory (5–10 minutes) and iii) mixing of blood with antigens (20 minutes). For both protocols, ground controls ran in parallel. Incubation temperature was kept constantly at 37 °C for all conditions.

#### In vitro challenging of the immune system using recall antigens in whole blood cell culture – delayed type hypersensitivity assay

*In vitro* DTH assays were performed as described previously with little modifications^47^. Briefly, whole blood aliquots (500 µl) were diluted under aseptic conditions with an equal volume of RPMI 1640 medium in assay tubes (round-bottomed 1.0 ml cryotubes; Nunc, Thermo Scientific). Used stimuli included LPS (5 µg/ml; Sigma-Aldrich, Steinheim, Germany), HKLM (5 µg/ml; InvivoGen Europe, Toulouse, France) and PWM (0.5 µg/ml; Sigma-Aldrich, Steinheim, Germany). Non-stimulated samples containing only medium served as control. Fully filled closed assay tubes were incubated for 6, 24 and 48 hours at 37 °C according to the protocols as explained above. At the end of incubation time, 100 μl from each supernatant was transferred into Eppendorf tubes and stored at −80 °C until cytokine measurement. The remaining blood dilution was conserved with Transfix (Biolegend, San Diego, CA, USA) and stored at 4 °C until further flow cytometric analysis.

#### Lymphocyte and monocyte population

Whole blood samples from individual donors were analysed for relative counts (%) of lymphocytes and monocytes using BD Multitest™ IMK Kit (BD Biosciences) according to the manufacturer’s instructions.

#### Activation state and inflammatory phenotype of monocytes and lymphocytes

From each DTH assay sample, 20 µl of blood dilution (per staining tube) was stained with fluorochrome-conjugated antibodies to detect expression of CD14 and CD16 as well as the activation markers TLR2, CD80, CD40, CD69 or HLA–DR respectively for analysis of monocytes. For T-lymphocytes, CD4 and CD69 or CD8 and CD69 were stained together with antibodies against CD28 or CD152. Antibody incubation was performed for 20 minutes at room temperature. With exception of TLR2-antibody (Miltenyi Biotec, Bergisch Gladbach, Germany), all used antibodies were obtained from BD Biosciences (Franklin Lakes, NJ, USA). For erythrocyte lysis, samples were incubated for 10 minutes with lysis buffer (BD FACS lysing solution, BD Biosciences Franklin Lakes, NJ, USA) and after a subsequent washing step, samples were analysed by flow cytometry (BD FACScan, Franklin Lakes, NJ, USA).

#### Cytokine measurement

The concentrations of IL-2, IL-6, TNF and IL-10 in plasma supernatants from DTH assay samples were quantified using the BD™ Cytometric Bead Array Kit (BD Biosciences, Franklin Lakes, NJ, USA) according to the manufacturer’s instructions. A standard curve was generated and concentrations in pg/ml were calculated from the *mean fluorescence intensity* (MFI) according to the curve.

### Statistics

Data analysis was performed with commercially available software (SPSS 20.0, IBM; SigmaPlot 12.5, Systat; Excel, Microsoft). For flow-chamber experiments, non-parametric Kruskal-Wallis test followed by Dunn’s test was applied for multiple comparisons since the data did not pass the normality test (Kolmogorov-Smirnov test). For whole blood data, we compared two groups and an unpaired student t-test was performed. Data are considered to be significant at a p < 0.05, p < 0.01 or p < 0.001 and are indicated as (*), (**) or (***) respectively. Results are expressed as means ± standard deviation (SD).

## Supplementary information

Flow-chamber at 1g

Flow-chamber at µg

Supplementary information
