# Supplementary material for: Cells´ Flow and Immune Cell Priming under alternating g-forces in Parabolic Flight
Source: Sci Rep. 2019 Aug 2;9:11276. doi: 10.1038/s41598-019-47655-x (PMC6677797; doi:10.1038/s41598-019-47655-x)
Supplement: Supplementary file 3 — Supplementary information [file 41598_2019_47655_MOESM3_ESM.pdf]

Cells´ Flow and Immune Cell Priming under alternating g-forces in Parabolic Flight

D. Moser[1#], S.J. Sun[2,3#], N. Li[2,3], K. Biere[1], M. Hoerl[1], S. Matzel[1], M. Feuerecker[1], J.-I. Buchheim[1], C. Strewe[1], C.S. Thiel[4,5], Y.X. Gao[2,3], C.Z. Wang[2,3], O. Ullrich[4,5], M. Long[2,3*], A. Choukèr[1*]

[#]contributed equally

Video 1 PBMCs passing a flow-chamber at 1g in PF Representative video (30 sec.), showing PBMCs at 1g. Exemplary cells, where rolling was clearly visible were marked chronologically with numbers 1-5.

Video 2 PBMCs passing a flow-chamber at µg in PF Representative video (23 sec.), showing PBMCs at the transition from hyper-g (1.8g) (first two seconds of video) to µg. At µg, no rolling cells were detectable.

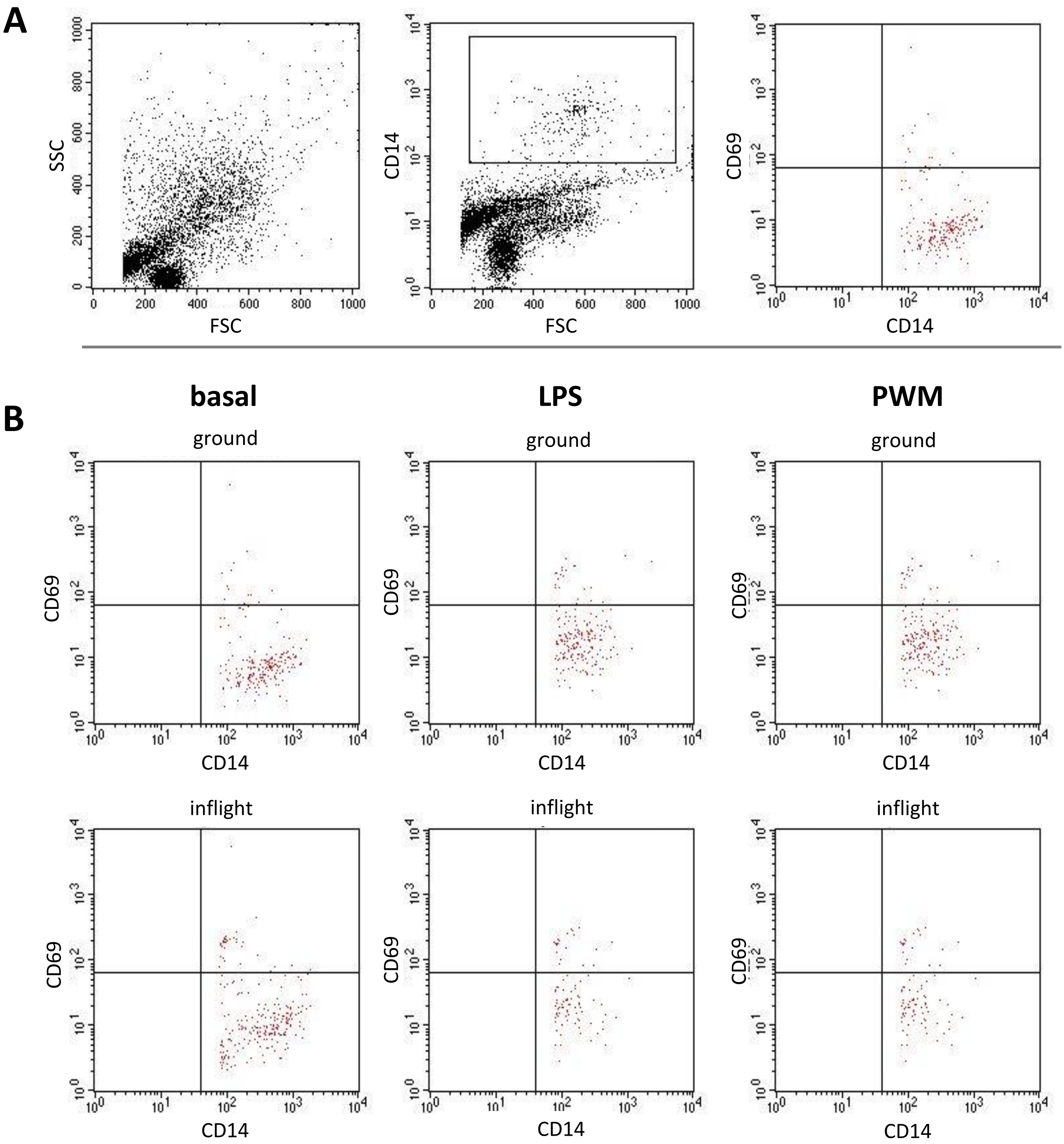

Fig. S1 Expression of activation marker CD69 on monocytes 24 hours after protocol "antigens during PF" After a total incubation time of 24 hours, samples were fixated for flow cytometric analysis of surface marker expression. Displayed are representative dot plots **A)** Gating strategy: From whole blood cell population (*Forward Scatter* (FSC)/*Side Scatter* (SSC) (left)), events positive for CD14 were identified (middle). CD14-positive events were gated and plotted for CD69. Events in the upper right quadrant represent CD14/CD69 double-positive cells (right). **B)** CD14/CD69 double-positive events for control (basal, left column), incubation with LPS (middle column) and PWM (right column) in ground (upper row) and inflight (bottom row) samples.

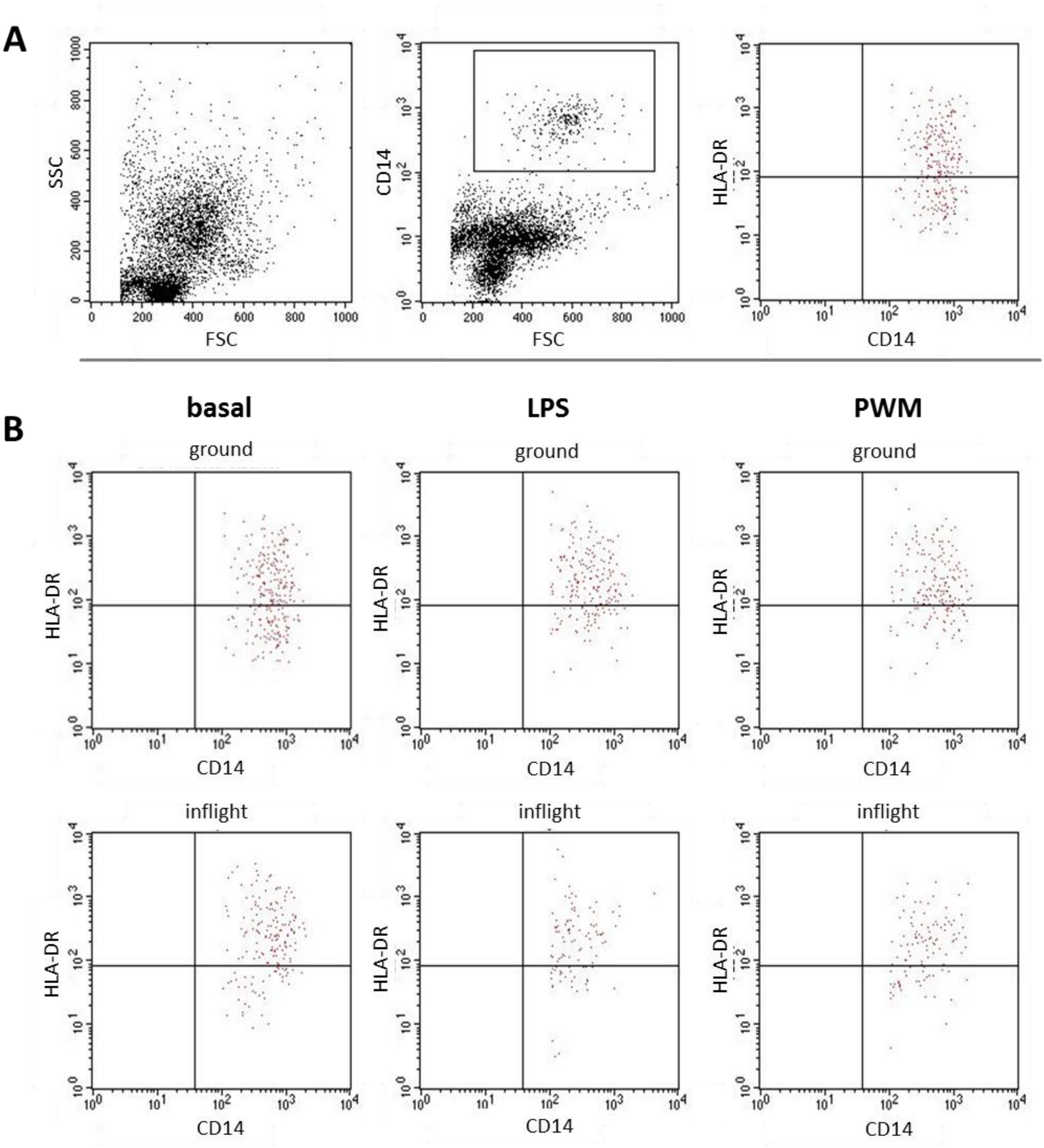

Fig. S2 Expression of activation marker HLA-DR on monocytes 24 hours after protocol "antigens during PF" A) Gating strategy: From whole blood cell population (FSC/SSC (left)), events positive for CD14 were identified (middle). CD14-positive events were gated and plotted for HLA-DR. Events in the upper right quadrant represent CD14/HLA-DR double-positive cells (right). **B)** CD14/HLA-DR double-positive events for control (basal, left column), incubation with LPS (middle column) and PWM (right column) in ground (upper row) and inflight (bottom row) samples.

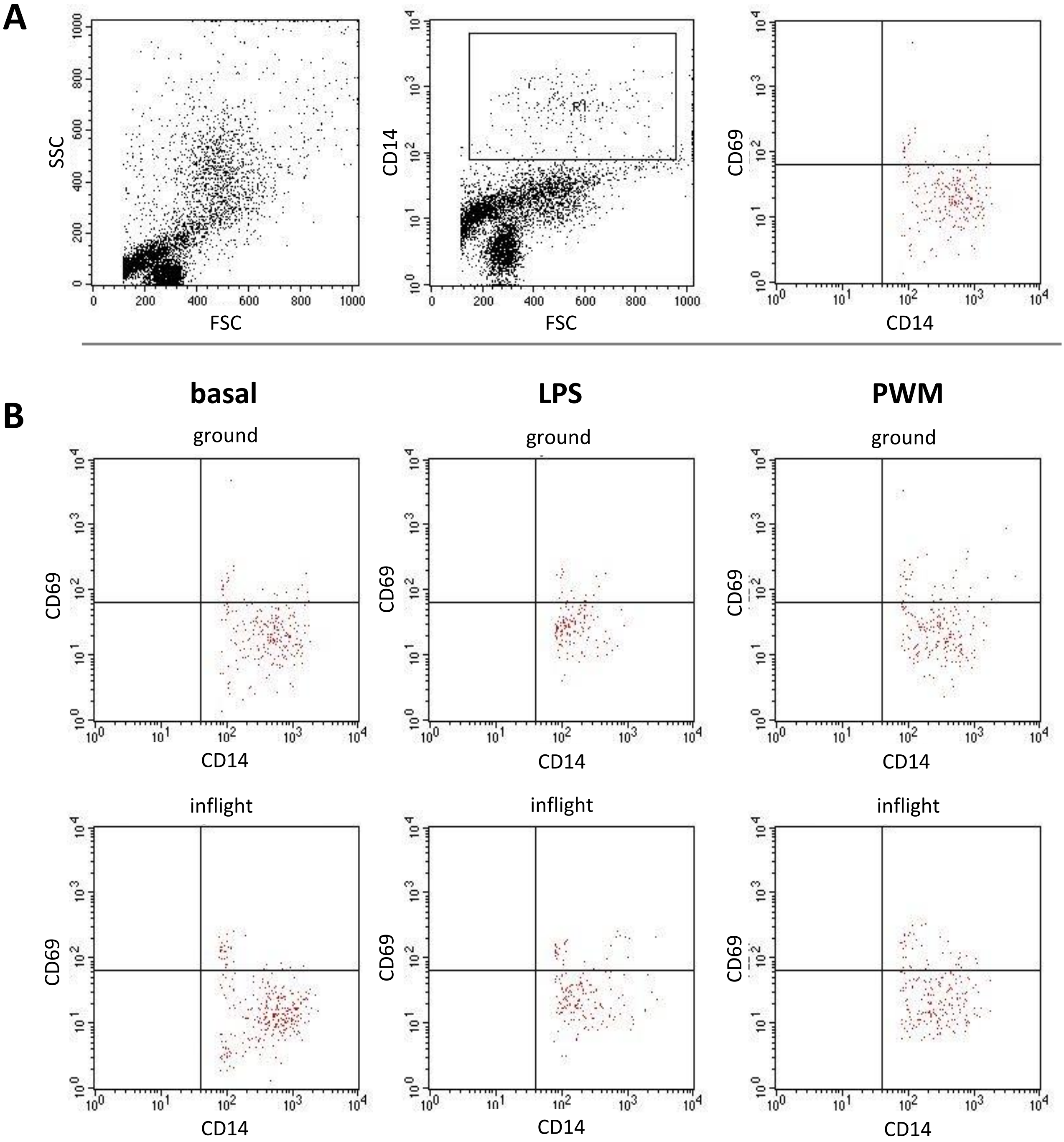

Fig. S3 Expression of activation marker CD69 on monocytes 24 hours after protocol "antigens after PF" After a total incubation time of 24 hours, samples were fixated for flow cytometric analysis of surface marker expression. Displayed are representative dot plots of flow cytometric analyses. **A)** Gating strategy: From whole blood cell population (FSC/SSC (left)), events positive for CD14 were identified (middle). CD14-positive events were gated and plotted for CD69. Events in the upper right quadrant represent CD14/CD69 double-positive cells (right). **B)** CD14 /CD69 double-positive events for control (basal, left column), incubation with LPS (middle column) and PWM (right column) in ground (upper row) and inflight (bottom row) samples.

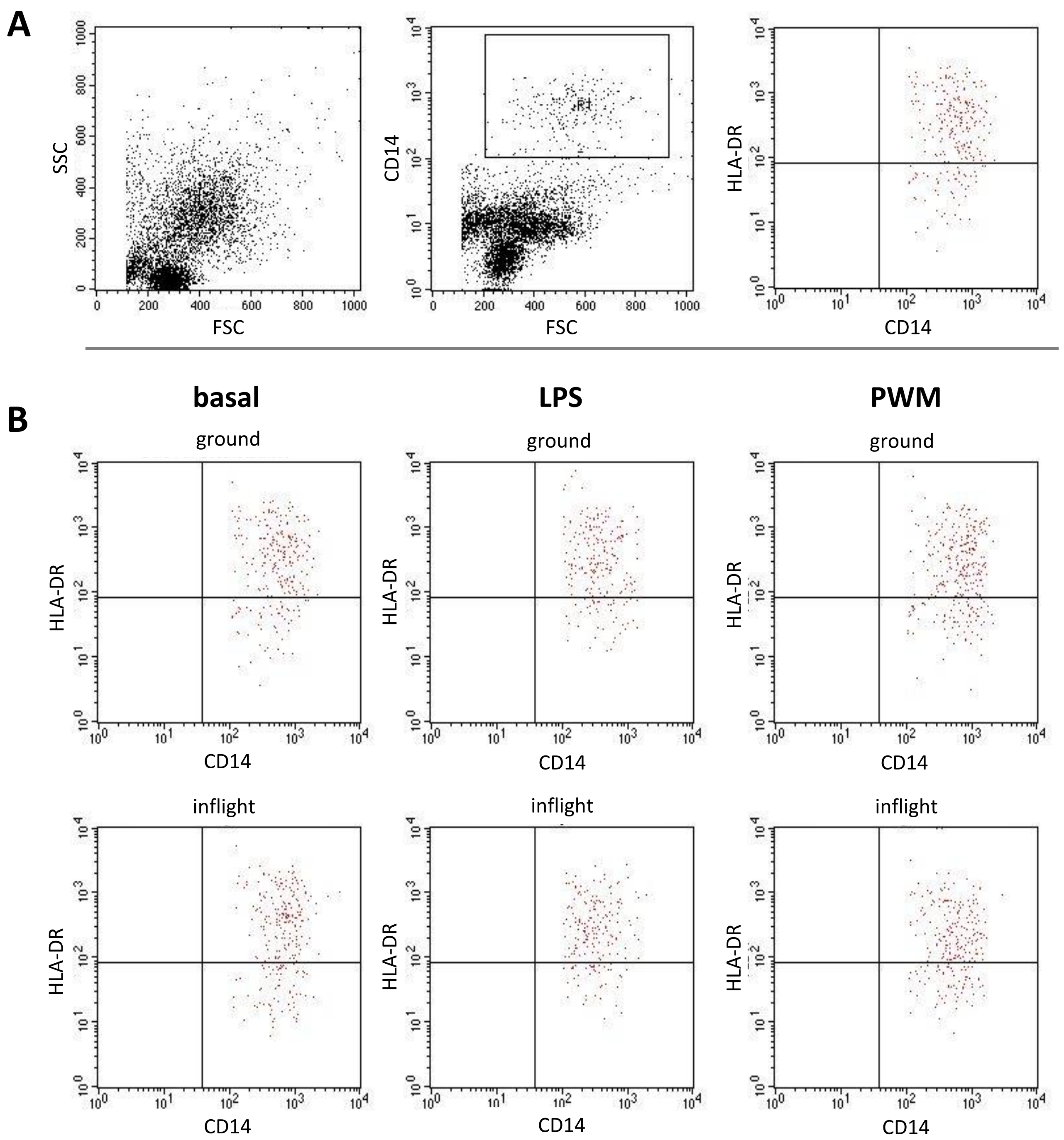

Fig. S4 Expression of activation marker HLA-DR on monocytes 24 hours after protocol "antigens after PF" A) Gating strategy: From whole blood cell population (FSC/SSC (left)), events positive for CD14 were identified (middle). CD14-positive events were gated and plotted for HLA-DR. Events in the upper right quadrant represent CD14/HLA-DR double-positive cells (right). **B)** CD14/HLA-DR double-positive events for control (basal, left column), incubation with LPS (middle column) and PWM (right column) in ground (upper row) and inflight (bottom row) samples.

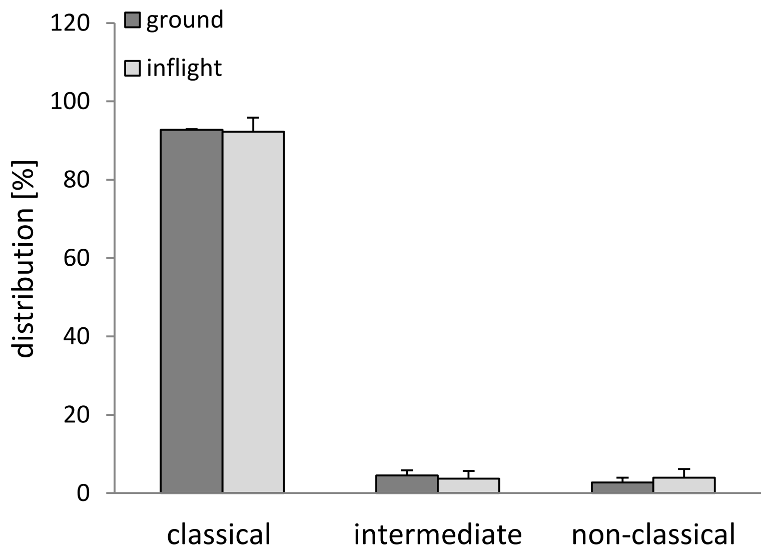

Fig. S5 Percentage distribution of monocyte subsets after PF Whole blood without antigen stimulus was exposed to PF. Classical monocytes: $CD14^{++}CD16^{-}$, intermediate monocytes: $CD14^{++}CD16^{-}$, non-classical monocytes: $CD14^{+}CD16^{++}$.
